# Denitrifying and diazotrophic community responses to artificial warming in permafrost and tallgrass prairie soils

**DOI:** 10.3389/fmicb.2015.00746

**Published:** 2015-07-21

**Authors:** Christopher R. Penton, Derek St. Louis, Amanda Pham, James R. Cole, Liyou Wu, Yiqi Luo, E. A. G. Schuur, Jizhong Zhou, James M. Tiedje

**Affiliations:** ^1^Department of Plant, Soil and Microbial Sciences, Center for Microbial Ecology, Michigan State UniversityEast Lansing, MI, USA; ^2^College of Letters and Sciences, Arizona State University, Polytechnic CampusMesa, AZ, USA; ^3^Institute for Environmental Genomics and Department of Microbiology and Plant Biology, University of OklahomaNorman, OK, USA; ^4^Department of Microbiology and Plant Biology, University of OklahomaNorman, OK, USA; ^5^Department of Biological Sciences, Center for Ecosystem Science and Society, Northern Arizona UniversityFlagstaff, AZ, USA; ^6^State Key Joint Laboratory of Environment Simulation and Pollution Control, School of Environment, Tsinghua UniversityBeijing, China; ^7^Earth Science Division, Lawrence Berkeley National LaboratoryBerkeley, CA, USA

**Keywords:** nifH, nirK, nirS, nosZ, denitrification, climate change, permafrost, warming

## Abstract

Increasing temperatures have been shown to impact soil biogeochemical processes, although the corresponding changes to the underlying microbial functional communities are not well understood. Alterations in the nitrogen (N) cycling functional component are particularly important as N availability can affect microbial decomposition rates of soil organic matter and influence plant productivity. To assess changes in the microbial component responsible for these changes, the composition of the N-fixing (*nifH*), and denitrifying (*nirS, nirK, nosZ*) soil microbial communities was assessed by targeted pyrosequencing of functional genes involved in N cycling in two major biomes where the experimental effect of climate warming is under investigation, a tallgrass prairie in Oklahoma (OK) and the active layer above permafrost in Alaska (AK). Raw reads were processed for quality, translated with frameshift correction, and a total of 313,842 amino acid sequences were clustered and linked to a nearest neighbor using reference datasets. The number of OTUs recovered ranged from 231 (NifH) to 862 (NirK). The N functional microbial communities of the prairie, which had experienced a decade of experimental warming were the most affected with changes in the richness and/or overall structure of NifH, NirS, NirK and NosZ. In contrast, the AK permafrost communities, which had experienced only 1 year of warming, showed decreased richness and a structural change only with the *nirK*-harboring bacterial community. A highly divergent *nirK*-harboring bacterial community was identified in the permafrost soils, suggesting much novelty, while other N functional communities exhibited similar relatedness to the reference databases, regardless of site. Prairie and permafrost soils also harbored highly divergent communities due mostly to differing major populations.

## Introduction

Microbial communities mediate positive feedback mechanisms to warming of the Earth's climate due to their decomposition of organic carbon (CO_2_, CH_4_) and nitrogen cycling (N_2_O). Community responses to warming such as changes in diversity, population size and shifts in functional groups ultimately impact carbon (C) and nitrogen (N) cycling pathways and rates. Microbially-mediated decomposition rates are impacted not only by climate but substrate quality and the composition of the microbial community (Cornelissen, 1996; Aerts, 1997; Berg and McClaugherty, 2007; Parton et al., 2007). For substrate quality, litter lignin and nitrogen content have strong effects on decomposition rates locally (Melillo et al., 1982; Taylor et al., 1989) and on the global scale (Cornwell et al., 2008), along with climate (Meentemeyer, 1978). However, the impact of exogenous N addition on decomposition has produced mixed results that are often ecosystem-dependent (Keller et al., 2010; Norris et al., 2012) with the general consensus that, particularly in sites with low N availability, decomposition is limited by nitrogen in the early phases (Hobbie et al., 2012). However, changes in plant community composition and thus species-specific litter chemistry (Norris et al., 2012), as a result of climate change (precipitation/temperature) or nutrient availability, may also impact the decomposer community and its responses to the addition or removal of N from the system. Lastly, by employing different methodological approaches such as qPCR and microbial community profiling techniques, temperature has been shown to significantly impact the size and structure of the denitrifying (Yergeau et al., 2007; Braker et al., 2010; Jung et al., 2011) and diazotrophic (Deslippe et al., 2005) communities.

In this study we utilized high-throughput targeted amplicon sequencing to assess changes in microbial communities involved in N cycling in two ecosystems, Alaskan permafrost and an Oklahoma tallgrass prairie, sites with a range of past and on-going complementary studies. The Carbon in Permafrost Experiment Heating Research (CiPEHR) project was established in Alaska in 2008 to examine the effects of experimental warming on permafrost biogeochemical cycling, and changes to the underlying microbial community. Winter warming (1.5–2.3°C to 40 cm depth) has resulted in a 10% increase in thaw depth and a 20% increase in gross primary productivity (GPP). Though no significant change in soil respiration was found during the growing season, a doubling of net CO_2_ loss was observed during the winter months (Natali et al., 2011). Winter warming also increased total plant N and since soil N mineralization is tightly coupled to plant N uptake, it was hypothesized that soil N availability was enhanced under warming (Natali et al., 2012). Experimental warming has also resulted in a shallower water table, increases in soil moisture and enhanced cellulose decomposition in the 0 to −10 cm soil depths (Natali et al., 2015). Fungal community composition was also shown to significantly differ between the soils positioned in the active layer, where seasonal thawing and freezing occurs, and the (frozen) permafrost layer that was first formed in the Pleistocene era (Penton et al., 2013b).

The tallgrass prairie ecosystem site was established in 1999. Experimental warming using infrared heaters has resulted in compositional and metabolic shifts within the soil microbial community, assessed through metagenomics, with significant enrichment of pathways relating to C degradation, CO_2_ production and N cycling (esp. denitrification). These changes were further linked to changes in aboveground plant productivity (Luo et al., 2014). Both GeoChip (Zhou et al., 2012) and shotgun metagenomic analyses (Luo et al., 2014) revealed significant differences in phylogenetic or functional gene richness and diversity with warming in addition to enrichment of metabolic pathways involved in N cycling. In contrast, the composition and diversity of fungal communities did not differ with warming (Penton et al., 2013b). Warming has also been shown to decrease plant tissue N concentration though total plant biomass N increased, due to higher biomass production (An et al., 2005). This was also linked to higher labile C and N, microbial biomass C and N, and indicated an increase in microbial C- and N- use efficiency (Belay-Tedla et al., 2009). Soil CO_2_ flux also increased with warming (Zhou et al., 2007).

Here we assessed changes in the diversity and composition of the N-cycling genes and their host microbial communities in these Alaskan permafrost and Oklahoma tallgrass prairie sites where experimental warming was underway for 1 year and one decade at the time of sampling, respectively. The overall goal of this project was to discern whether molecular tools are able to provide insight into the sensitivity of N-cycling communities to warming, regardless of site. The observed changes in N availability and cycling at these sites with warming led us to hypothesize that significant alterations in the N cycling community compositions would be driving these process-level differences. In order to address this question, targeted high-throughput amplicon sequencing of portions of the *nirK, nirS*, and *nosZ* genes, functional gene markers for denitrifying bacteria as well as of *nifH* which encodes a subunit of the N_2_-fixing nitrogenase complex in diazotrophic bacteria, was performed. Our results suggested that N functional gene-harboring bacterial richness and overall composition were most affected in the tallgrass prairie soils after 10 years of warming. After only 1 year of warming the Alaskan permafrost N processing communities were less affected. The *nirK* harboring bacterial community: (i) was found to be the most sensitive to warming, (ii) was the only N functional gene community significantly altered by warming in the permafrost samples, and (iii) contained highly divergent NirK sequences in the permafrost samples.

## Methods

### Site description

The two experimental warming sites in this study are subjects of ongoing studies examining changes in biogeochemical pools and fluxes; plant, fungal and bacterial community structures; soil metagenomes and metatranscriptomes and other impacts related to short-term and long-term experimental warming. The Oklahoma (OK) samples originated from the unclipped plots at the Great Plains Apiaries site (34°58′54″N, 97°31′14″W). Initiated in November 1999, its purpose was to determine ecosystem (plant and soil) responses to experimental warming and is a subject of continuing research (Luo et al., 2001, 2014; Wan et al., 2002a,b; Zhang et al., 2005; Zhou et al., 2007, 2012; Jia et al., 2014; Xu et al., 2014). The grassland is dominated by C_3_ forbs *Xanthocephalum texanum* and *Ambrosia psilostachyia* and the C_4_ grasses *Sorghastrum nutans, Schizachyrium scoparium* and *Eragrostis curvula*. The control plot (C) contained a dummy heater to simulate the shading effect while experimental warming (T) was performed with an infrared heater. Mean soil temperatures in the warming plot were increased by 1.8–2.7°C to a depth of 10 cm. Soil cores were collected in October 2009 from the surface layer (0–15 cm) of six replicates in control and six in warming plots.

Alaskan (AK) permafrost samples originated from the Carbon in Permafrost Experimental Heating Research project (CiPEHR). Located near Denali National Park and Preserve near Eight Mile Lake, Alaska (63°52′59″N, 149°13′32″W), the site was established in September 2008. The overarching goal of this site is to determine the effects of soil and permafrost warming on tundra ecosystems (Natali et al., 2011) and is a subject of ongoing research, as noted prior. Accumulating snow due to snow fences passively increased the winter soil temperatures by 1.5°C (over a depth of 5–40 cm) (Natali et al., 2011) and resulted in thawing of the near-surface permafrost layers. Accumulated snow was removed in the spring to reduce the impact of excess snowmelt at the site. The sites are dominated by *Eriophorum vaginatum* and *Vaccinium uliginosum* along with other vascular plants (Luo et al., 2001; Schuur et al., 2007, 2009; Natali et al., 2011). Soil cores were taken in May 2009 up to a 65 cm depth from control (C) and winter warming (T) plots after 1 year of warming. The cores were sliced into 10 cm depth increments while the active layer was still frozen and the depth to the permafrost layer was determined in the field. For both sites samples remained frozen until DNA extraction using the mechanical lysis method (Zhou et al., 1996) without the removal of plant roots. In total, 12 samples per treatment were analyzed from the 0 to −45 cm depths, corresponding to soils above the permanently frozen permafrost depth (−50 cm) and average water table depth (−25 cm) (Natali et al., 2011).

### Amplification and sequencing

All PCR amplifications for *nifH, nirS, nirK*, and *nosZ* were performed in quadruplicate using primers tagged with multiplex identifier (MID) sequences. Primer sequences and conditions are noted (Supplementary Table 1). Amplicons were sequenced using the 454 Titanium pyrosequencing platform at the Utah State University Center for Integrated Biosystems. Raw reads were deposited in the European Nucleotide Short Read Archive (SRA) under study accession PRJEB8005, sample accession numbers ERS629288-ERS629367. Nucleotide sequences were processed using the Ribosomal Database Project (RDP) functional gene (FunGene) pipeline (Fish et al., 2013; http://fungene.cme.msu.edu). Chimeric sequences were removed using UCHIME 6.0 in de novo mode. The filtered sequences were translated to protein and frameshift-corrected using the RDP DNA-protein alignment tool FrameBot (min length = 80 a.a., identity cutoff = 80%) (Wang et al., 2013) and the corresponding FunGene database as a reference. Amino acid sequences were then aligned and clustered using complete linkage clustering at the respective amino acid dissimilarity threshold determined for each gene (NifH, NirS 5%; NirK NosZ 3%) (Supplementary Figure 1). Sequences were randomly re-sampled for each gene to between 1053 and 3800 sequences per sample (Supplementary Table 2). Representative minimum sum of square distances sequences for each cluster were generated and used for BLASTp (closest-match) against reference databases generated from the RDP Fungene database. For *nifH*, representative sequences were subjected to BLASTp against a *nifH* database where the extracted protein region that corresponds to that amplified by the primers was used (augmented Zehr-set; Wang et al., 2013).

### Statistical analyses

Raw sequence abundances were normalized by Hellinger transformation (square root of relative abundance) and Bray-Curtis (+1) dissimilarity matrices were constructed using the PRIMER-6 package (Clarke and Warwick, 2001) (Primer-E Ltd, 239 Plymouth, U.K.). Ordinations were produced using permutated non-metric multidimensional scaling (nMDS) and significant differences among treatments tested using permutational analysis of variance (PERMANOVA) (Anderson, 2001). The effect of replicate dispersion on PERMANOVA results was tested using permutational analysis of multivariate dispersions (PERMDISP) and the individual OTUs that contributed to the majority of the Bray-Curtis dissimilarity were obtained using similarity percentage analysis (SIMPER) (Warwick et al., 1990). Margalef's richness (d) and Pielou's Evenness were calculated using PRIMER-E and significant differences among treatment groups tested using ANOVA analyses (Minitab 16, Minitab Inc., USA). Comparative tests on environmental and community similarity matrices were performed using the function RELATE. Gene-based resemblance matrices were compared to those created from log-transformed, normalized Euclidean distance matrices using the function BEST.

## Results

The amino acid dissimilarity value at which to cluster was determined by analyzing the number of OTUs recovered at 1–15% amino acid dissimilarity for an inflection point for each gene (Supplementary Figure 1), with the exception of NifH that was pre-determined at 5% dissimilarity (Wang et al., 2013). Cluster analysis was based on 3% amino acid dissimilarity for NirK and NosZ while NirS was based on 5%. Overall, the fraction of sequences with similarity to a reference protein sequence above the FrameBot cutoff was lower in the AK vs. Oklahoma (OK) samples for all functional genes (Supplementary Table 2). For NirS, NirK and NosZ the forward and reverse translated reads were clustered separately and the resulting OTU tables were combined into one data matrix for downstream analyses while NifH were unidirectional reads. The following gene-specific results involve two comparisons: (i) the effect of warming within each site and (ii) the differences between sites (AK and OK), regardless of treatment (both control and warming treatments are combined for these comparisons).

### NifH

For *nifH*, a total of 232 OTUs were generated at 5% amino acid dissimilarity that represented closest BLASTp matches to 81 unique taxa from the reference dataset. Both gene richness and evenness were significantly increased in the warming treatment in OK, though the increase was not significant in AK (Table 1). PERMANOVA results showed no significant warming effect on the diazotroph community structure in both OK and AK (Table 2). Two samples were identified as outliers in OK, each within the warming treatment. Both were distant from both AK and OK, with an average of 25.1% similarity to all other samples. PERMANOVA was not possible after the removal of these samples although ANOSIM results showed a non-significant differentiation with warming (Global *R* = 0.1, *p* = 0.21). PERMANOVA revealed no significant community difference between the organic (surface) and mineral (variable depth) layers in AK (*F* = 2.25, *p* = 0.072), but with significant differences between the samples taken above and below the average water table depth (*F* = 6.01, *p* = 0.004).

**Table 1:** **Margalef's species richness (J') and Pielou's evenness in Oklahoma (OK) and Alaska (AK) control (C) and artificial warming (T) samples**.

	**Margalef richness (d)**				**Pielou's evenness (J')**			
	**OK-C**	**OK-T**	**AK-C**	**AK-T**	**OK-C**	**OK-T**	**AK-C**	**AK-T**
*nifH*	**8.31**^*b*^	**10.26**^*a*^	8.58^c^	9.00^c^	**0.552**^*b*^	**0.581**^*a*^	0.516^c^	0.575^b^
*nirS*	**38.59**^*b*^	**51.73**^*a*^	22.83^c^	21.36^c^	**0.759**^*b*^	**0.848**^*a*^	0.729^c^	0.709^c^
*nirK*	53.38^ab^	57.44^a^	**65.68**^*a*^	**41.82**^*b*^	0.968^a^	0.966^a^	0.932^b^	0.924^b^
*nosZ*	51.41^ab^	64.55^a^	30.71^c^	33.81^bc^	0.958^ab^	0.967^a^	0.935^c^	0.945^bc^

*Bolded pairs highlight significant differences between control and artificial warming samples. Superscript letters indicate ANOVA grouping. nd, not determined*.

**Table 2:** **Overview of PERMANOVA and permutational dispersion (PERMDISP) results for the indicated functional gene containing bacterial community addressing warming treatment effects of the Oklahoma (OK) and Alaska (AK) sites and the comparison of AK vs. OK**.

	**Oklahoma**			**Alaska**			**AK-OK**		
	**F**	**P**	**Disp-P**	**F**	**P**	**Disp-P**	**F**	**P**	**Disp-P**
*nifH*	2.00	0.209	0.06	0.652	0.629	0.67	**2.56**	**0.001**	0.37
*nirS*	0.995	0.270	0.30	0.633	0.841	0.53	**8.87**	**0.001**	0.74
*nirK*	**50.50**	**0.001**	0.50	**2.05**	**0.048**	0.47	**8.91**	**0.001**	0.58
*nosZ*	**2.30**	**0.004**	0.64	0.580	0.857	0.68	**6.89**	**0.001**	**0.01**

*Significant comparisons are bolded to highlight*.

**Figure 1:**
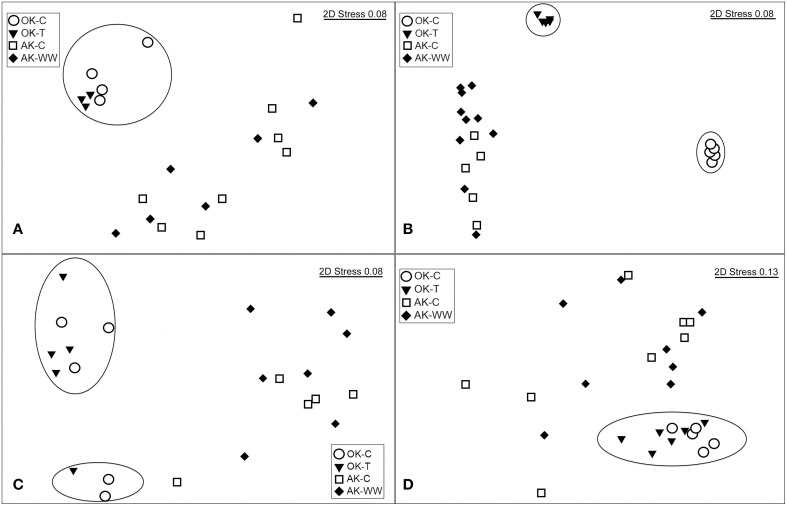
**nMDS ordinations based on Bray-Curtis dissimilarity matrices for (A) NifH, (B) NirK, (C) NirS and (D) NosZ for the Oklahoma control (OK-C) and warming (OK-T) and Alaska control (AK-C) and warming (AK-WW) sites**. For NifH, the two outliers were removed for visualization of site relationships.

Significant overall community differences were identified according to site (AK vs. OK), without regard to treatment (Table 2). According to NMDS ordination, OK samples were tightly grouped with a contrasting large dispersion of AK samples (Figure 1A). Both AK and OK diazotrophic communities were weighted heavily toward a few OTUs, which contributed to the lowest evenness of all functional genes. Average BLASTp identities to the reference database were 89.4 ± 7.3% in OK and 89.0 ± 5.0% in AK. One OTU (7V1S) that grouped with Alpha- and Betaproteobacteria was present in high abundance in both AK and OK, with an average of 33.7 and 17.7% relative abundance, respectively (Supplementary Figure 2). This OTU was most closely matched to *Rhizobium* sp. at 93.5% amino acid identity. A second OTU (6U2R), also assigned to *Rhizobium* sp., was most associated with AK at 19.4% relative abundance but only 1.5% in OK. In total, more OK than AK NifH sequences exhibited similarities to the Deltaproteobacteria while there appeared to be a somewhat larger diazotrophic population in AK most closely identified to the Verrucomicrobia at 85.5–96.3% amino acid similarity. Overall, OTUs with a relative abundance >1.0% had significantly higher percent identities (92.2 ± 0.5%) to the reference database (*t*-test, *p* = 0.01) than the more rare OTUs (88.8 ± 0.7%).

### NirK

For *nirK*, FrameBot translation and frameshift correction required a decrease in the threshold identity cutoff to the FrameBot reference database to 20%. This was due to the exclusion of *Bradyrhizobium*-like sequences identified through BLASTn using the NCBI non-redundant (nr) database. These sequences were <40% amino acid identity to the FrameBot reference training set that consisted of 77 reference sequences. The nirK reference dataset for best-match analysis using BLASTp was created using the FunGene nirK database set at a minimum score of 220, HMM coverage of 80 and filtered to 381 sequences. At 3% amino acid dissimilarity, a total of 1690 OTUs (828 forward, 862 reverse) that represented 126 unique closest-match taxa were identified (Supplementary Table 2). Among all genes, NirK richness was highest in AK, and differed significantly between the control and warming sites in AK but not OK (Table 1). PERMANOVA showed no significant community difference between the organic and mineral layers in AK (*F* = 1.13, *p* = 0.317) as well as between samples originating from above and below the water table depth (*F* = 1.41, *p* = 0.143).

**Figure 2:**
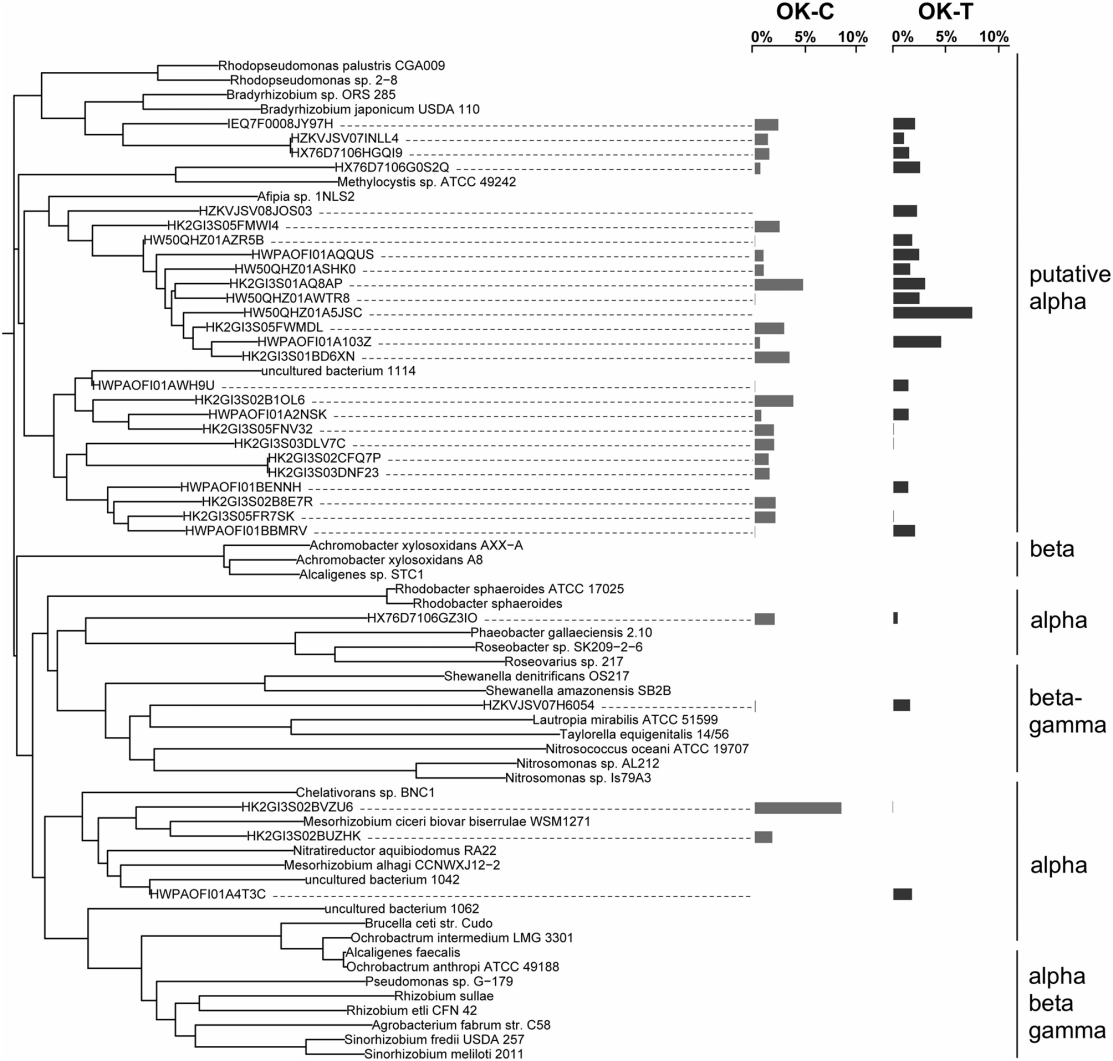
**NirK neighbor-joining tree based on amino acid sequences showing the most abundant 32 OTU representative sequences comparing Oklahoma control (OK-C) to warming (OK-T) samples with reference sequences**. The OTUs shown contain 48.3% of sequences in OK-C and 43.4% of the sequences in OK-T. The average relative abundances are represented by bar graphs to the right of the OTU. The first column corresponds to OK-C, the second to OK-T.

**Figure 3:**
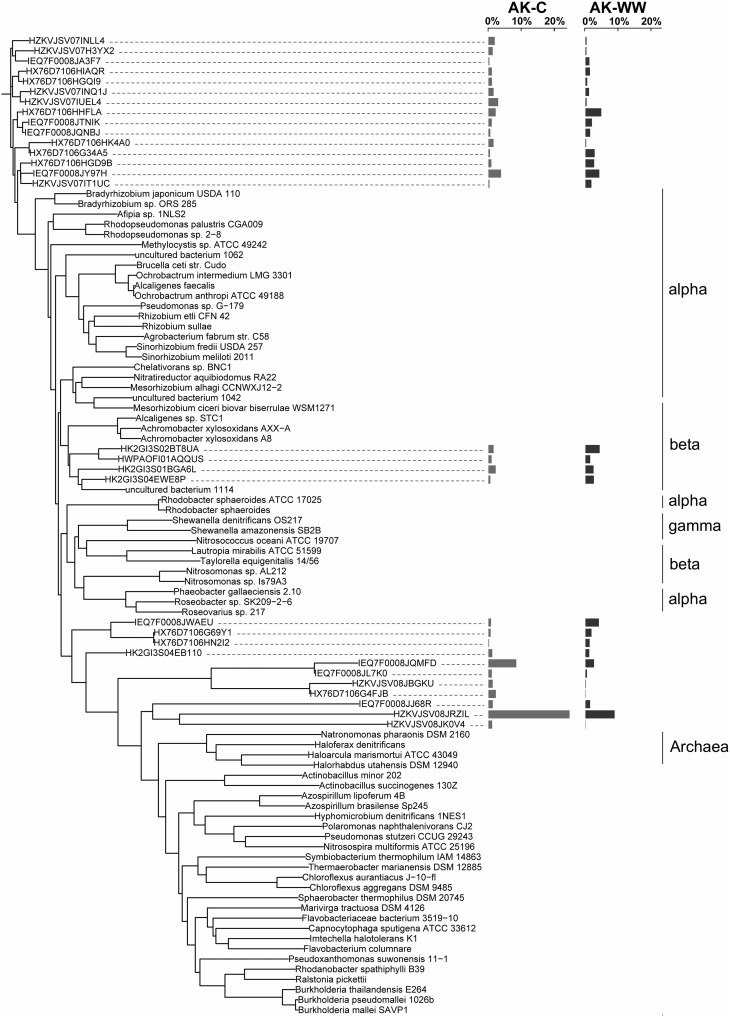
**NirK neighbor-joining tree based on amino acid sequences showing the most abundant 33 OTU representative sequences from Alaska control (AK-C) and warming (AK-WW) with reference sequences**. The OTUs shown contain 78.0% of sequences in AK-C and 72.0% of the sequences in AK-WW. The average relative abundances are represented by bar graphs to the right of the OTU. The first column corresponds to AK-C, the second to AK-WW.

The overall bacterial *nirK* gene-containing community structure was significantly different with warming in both OK and AK (Table 2). NMDS ordination showed a clear distinction in OK, less so in the AK samples (Figure 1B). Similarity percentage analysis (SIMPER) for the warming effect revealed that 69 OTUs contributed to 50% of the Bray-Curtis dissimilarity in OK while 28 OTUs contributed in AK. Trees illustrating the significant change in the overall *nirK*-containing bacterial community with warming showed that the difference was mostly due to changes in relative abundance, not presence-absence (Figures 2, 3). In relation to OK warming, there were a few exceptions including OTU BVZU6 (98.1% a.a. identity to *Mesorhizobium amorphae*) at an average of 8.6% (OK-C) and 0.0% (OK-T) relative abundance and OTU A5JSC (distantly related to *Rhodopseudomonas palustris* at 78.3% identity) at 0.0% (OK-C) and 7.4% (OK-T) relative abundance (Figure 2). A deep branching clade consisting of 7 OTUs (OTUs JQMFD-JK0V4) in the AK warming comparison (Figure 3) exhibited <33% amino acid similarity to a nearest uncultured bacterium in the dataset, indicating a potentially abundant unknown component in these soils.

For AK vs. OK, a significant site influence on total community structure was identified without regard to treatment (Table 2) (Figure 1B). Average percent identities of the OTU representative sequences to the reference database indicated that the *nirK*-harboring communities unique to AK are largely unknown (36.5 ± 16.3% ID), compared to the higher identifies in OK (81.7 ± 7.3% ID). Within the AK site, 26.2% of sequences in 27 OTUs had no best match in the reference database and thus identity was not reported. In comparison, only one sequence in OK did not match to the database. A total of 148 OTUs accounted for 50% of the Bray-Curtis dissimilarity. In OK, these OTUs exhibited the highest amino acid similarity to *Mesorhizobium, Rhodopseudomonas, Nitratireductor*, and *Methylocystis*. The low identities in AK present an obstacle to establishing any relationship to a closest database match. Sites were differentiated in a large part due to presence/absence and were grouped in specific clades specific to each site (Supplementary Figure 3).

### NirS

For *nirS*, 34.7 and 41.4% of NirS sequences failed FrameBot (length 80, identity = 40) in the OK and AK samples, respectively. This was due to low amino acid identity (21–39%) to the reference database consisting of 210 sequences retrieved from FunGene with a minimum HMM coverage of 80% and minimum score of 500. A subset of the FrameBot failed sequences was subjected to BLASTn against the nr NCBI database. The majority of hits were to non-contiguous *nirS* gene regions and non-target genes. The non-target near-full length hits include those with putative functions to pyruvate dikinases, hypothetical proteins, phosphoenolpyruvate carboxykinases, cytochrome p460, CoA substrate specific enzyme activases and quinolinate synthetases, among others. As such, the FrameBot filter was set to 40% amino acid identity and the failed sequences were not considered for random re-sampling.

A total of 1245 OTUs were identified at 5% amino acid dissimilarity from a total of 52,580 passing reads (Supplementary Table 2). *NirS*-harboring bacterial community richness and evenness were significantly higher in the OK warming vs. control samples with no significant change observed in AK. The overall *nirS*-harboring bacterial community structure was not altered significantly with warming in either OK or AK (Table 2). PERMANOVA showed no significant community difference between the organic and mineral layers in AK (*F* = 0.938, *p* = 0.545) or with position above or below the water table depth (*F* = 1.23, *p* = 0.242). Comparing sites, OK NirS richness and evenness at OK were significantly higher than those at AK samples and a significant difference in community structure was observed between sites (Table 1). Compared to other genes, OK samples were the least tightly grouped but were clearly distinct from the AK samples (Figure 1C). Average BLASTp identities of OTU representative sequences to the reference database were nearly identical in both OK (76.2 ± 6.3%) and AK (76.0 ± 7.3%). The mostly low percent identities in AK do not allow for a reasonable relationship to the closest reference database match for any particular OTU. Differences in community structure between sites were attributed to changes in relative abundances in the most abundant OTUs (Supplementary Figure 4). OTUs tended to group together with no anchored reference sequence, indicating potentially novel clades.

### NosZ

**Figure 4:**
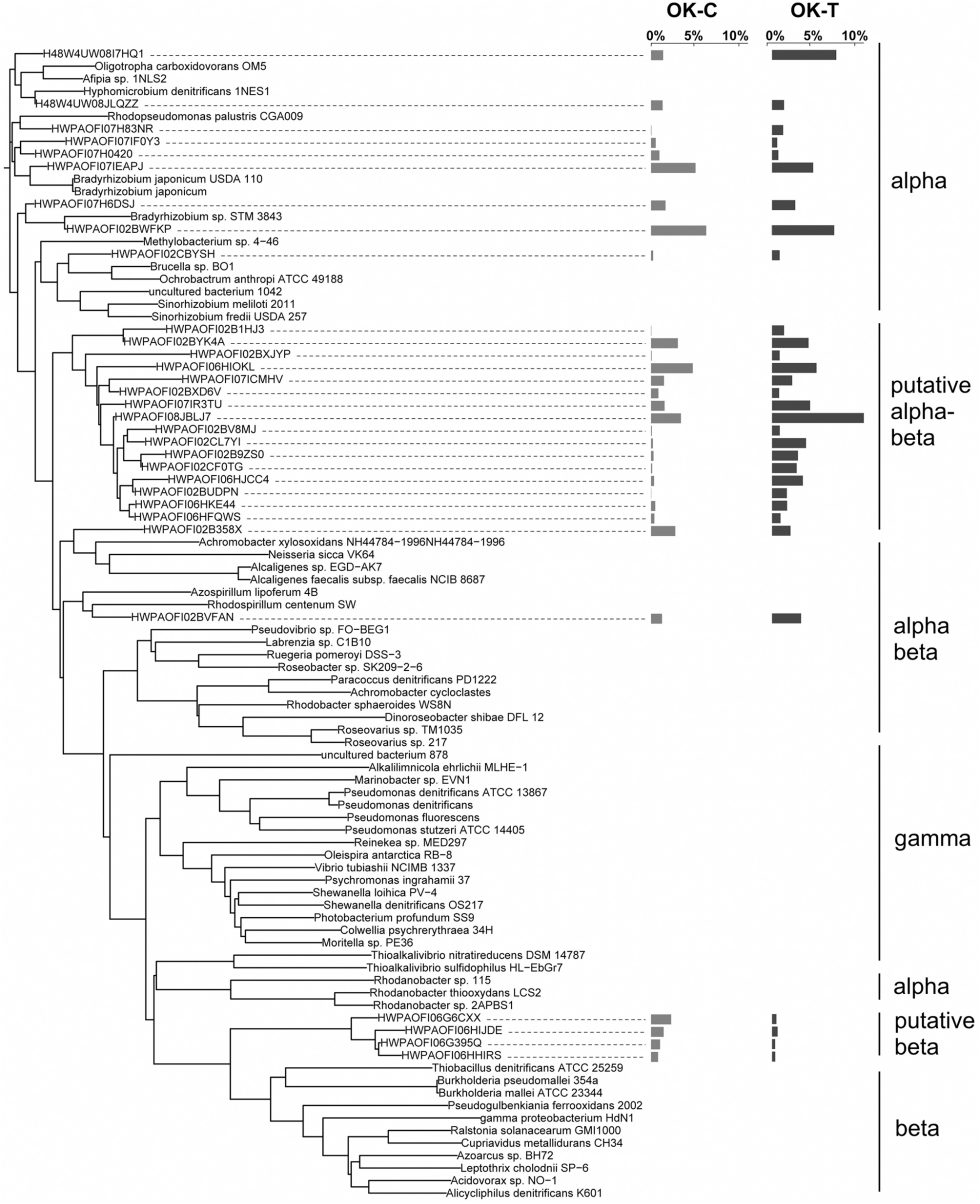
**NosZ neighbor-joining tree based on amino acid sequences showing the most abundant 31 OTU representative sequences from Oklahoma control (OK-C) and warming (OK-T) with reference sequences**. Tree is based on forward reads only. The OTUs shown contain 41.6% of sequences in OK-C and 86.9% of the sequences in OK-T. The average relative abundances are represented by bar graphs to the right of the OTU. The first column corresponds to OK, the second to AK.

*NosZ* sequences were translated through FrameBot at a minimum length of 80 amino acids at a 40% minimum similarity using a reference dataset of 160 NosZ sequences. A total of 1144 NosZ clusters were identified following clustering at 3% amino acid dissimilarity. The NosZ reference database consisted of 426 amino acid sequences parsed from FunGene with a minimum HMM coverage of 80 and minimum score of 360. A total of 52 unique closest-match bacteria were identified after BLASTp analysis of OTU representative sequences. Although there was a trend of increasing richness and evenness with warming in both OK and AK, these differences were not significant (Table 1). Warming significantly affected the overall *nosZ*-harboring bacterial community structure in OK but not in AK. Tree analyses of the most abundant OTUs showed that changes in relative abundances were responsible for the observed community differences (Figure 4) with a large, independent clade accounting for the majority of sequences (OTUs 1HJ3–FQWS). PERMANOVA showed no significant community difference between the organic and mineral layers in AK (*F* = 1.53, *p* = 0.162) though a significant difference based on position in relation to water table depth was identified (*F* = 3.56, *p* = 0.002).

The *nosZ*-harboring communities were different between OK and AK (Table 2), regardless of treatment, though a significant difference in dispersion (PERMDISP) may have influenced the PERMANOVA results. OK sites were grouped tightly and independent of the AK sites (Figure 1D). Percent amino acid identities to the reference database were low and nearly identical in both OK (53.3 ± 6.0%) and AK (54.0 ± 5.2%), indicating numerous, potentially novel bacteria. As in the OK warming contrast, the difference in *nosZ*-harboring communities between AK and OK were due to changes in relative abundances with large clades distant from reference sequences (Supplementary Figure 5).

### Edaphic factors and community structure

Bulk density (BD), percent nitrogen (%N) and percent carbon (%C) did not differ significantly with the warming treatment in Alaska (ANOVA, *p* > 0.05). However, there was a significant difference between the organic and mineral layers (Supplementary Table 3) with the organic layers having lower BD and higher %N and %C. In the tallgrass prairie (OK) there were no significant differences with warming for moisture, pH, NO^−^_3_, NH^+^_4_, total nitrogen, total carbon or percent organic matter. The PRIMER function RELATE, using all environmental data, showed that within the Alaskan permafrost, *nifH* and *nirK* harboring bacterial community structures were significantly correlated to the environmental matrices containing bulk density while *nosZ* was marginally correlated to percent carbon (Table 3). In the AK samples, bulk density was the highest contributor to BEST Spearman rank correlations for most genes. However, correlation was only substantial for NirK (BD, ρ = 0.205, *p* = 0.05). NosZ was again best correlated to %C, though not significantly (ρ = 0.213, *p* = 0.15). In the OK samples only NifH had a significant correlation to pH and total nitrogen (TN) combined (ρ = 0.723, *p* = 0.05) whereas NirS was correlated to the combination of NH^+^_4_, TN, and total carbon (TC) (ρ = 0.593, *p* = 0.05).

**Table 3:** **RELATE statistics (rho and ***p***-values) from comparing resemblance matrices between each gene and environmental variables in the tallgrass prairie (OK) and permafrost samples (AK)**.

		**OK**							**AK**		
		**Moisture**	**pH**	**NO^−^_3_**	**NH^+^_4_**	**TN**	**TC**	**OM**	**BD**	**%N**	**%C**
NifH	rho (ρ)	−0.13	0.30	0.26	0.13	**0.54**	0.49	0.16	**0.24**	0.05	0.17
	P	0.72	0.17	0.17	0.26	**0.03**	0.43	0.24	**0.04**	0.30	0.11
NirK	rho (ρ)	−0.06	0.05	0.03	0.10	0.04	−0.06	−0.06	**0.21**	0.07	0.13
	P	0.66	0.34	0.37	0.23	0.40	0.64	0.71	**0.04**	0.21	0.11
NirS	rho (ρ)	0.08	0.54	0.17	**0.43**	0.04	0.26	0.25	−0.13	0.13	−0.04
	P	0.26	0.07	0.81	**0.01**	0.32	0.08	0.10	0.78	0.13	0.61
NosZ	rho (ρ)	0.06	0.19	0.12	−0.02	−0.22	0.05	0.05	0.09	0.11	0.21
	P	0.35	0.19	0.26	0.49	0.88	0.37	0.39	0.25	0.16	0.07

*P-values less than 0.10 are bolded. TN, total nitrogen; TC, total carbon; OM, organic matter; BD, bulk density*.

## Discussion

### Warming effects on diversity and structure

We examined the diversity and composition of denitrifying and nitrogen-fixing microbial communities under experimental warming in Alaskan permafrost and a tallgrass prairie after 1 and 10 years of warming, respectively. Experimental warming in the Oklahoma tallgrass prairie surface soils (0 to −10 cm), where warming was initiated over a decade ago, resulted in significantly higher richness and evenness of the *nifH* and *nirS* harboring communities and significant shifts in the overall *nirK* and *nosZ* community structures (Tables 1, 2). This increase in richness differs from the results of Sheik et al. (2011) where decreased overall microbial community diversity was associated with warming at the same tallgrass prairie site (under normal precipitation) after 4 years of warming. In addition, fungal communities were found not to differ significantly with warming after a decade of warming (Penton et al., 2013b). However, GeoChip (Zhou et al., 2012) and shotgun metagenomic analyses (Luo et al., 2014) at these identical sites after 10 years of warming detected significant phylogenetic shifts, differences in functional gene richness and diversity and enrichment of metabolic pathways involved in N cycling and CO_2_ production.

After only 1 year of warming in Alaska, only the *nirK* harboring community was significantly altered with a corresponding decrease in richness (Tables 1, 2). In agreement with our overall trends within the permafrost samples, lower *nirK* and *nirS* and higher *nosZ* diversity have been identified in cryoturbated peat vs. unturbated peat soils (Palmer and Horn, 2012). It has been proposed that *nirS* (heme) bearing denitrifiers are better adapted to waterlogged soils (Kim et al., 2008; Petersen et al., 2012) while *nirK* (Cu) denitrifiers appear to be more associated with drier soils (Dandie et al., 2008; Bremer et al., 2009). While *nirK* was the most impacted by warming, the question remains as to whether they are the dominant denitrifiers in these soils and thus community changes would more likely be reflected through the changes in process rates.

Thus, overall, the *nirK*-containing denitrifiers were the most sensitive to warming, with significant community shifts at both sites. Linking these changes in diversity or functional community structure to process-level outcomes is difficult across systems, though correlations between qPCR quantities of genes for nitrite reduction (*nirK*) and N_2_O reduction (*nosZ*) and potential denitrification rates was observed across Alaskan ecosystem types (Petersen et al., 2012). In support of our findings in Alaska, *nirK* was found to be the most sensitive N cycling gene to warming and N addition, with increased abundances with warming in Antarctic soils (Jung et al., 2011).

### Differences among sites

As a proxy for contrasting soil physicochemical properties, climate and other factors (e.g., different plant chemical and physical characteristics, soil micro- macrofauna), the Oklahoma tallgrass prairie and Alaskan permafrost soils harbored distinctly different N-gene-harboring bacterial communities, regardless of warming treatment (both control and warming were combined for these comparisons) or length of treatment. In general there was lower gene richness in the permafrost, perhaps due to the selective pressures of low temperatures and anaerobiosis. With our targeted sequencing approach we found that presence-absence contributed more to the significant denitrifying community differences between these two sites, as compared to the observed within-site differences that were driven by differences in relative abundances. However, there were instances where major populations were shared among sites, possibly indicative of generalists that are viable in both locations. Other studies targeting denitrification genes have shown that few genotypes are shared between disparate locations (Braker et al., 2000, 2001; Scala and Kerkhof, 2000).

The tallgrass prairie diazotrophic (N-fixing) communities exhibited the lowest within-site variability of any N cycling genes. This could perhaps be due to less robust differences in small-scale soil and plant cover heterogeneity, some of the multiple factors that have been found to influence diazotrophic communities. These tend to be habitat-specific and related to plant cover, soil texture and chemistry, which in turn influence community composition across scales (Zehr et al., 1998; Shaffer et al., 2000; Poly et al., 2001). Conversely, the large within-site variability observed in the permafrost samples is likely due to the range of sampled depths and thus additional influences such as water availability, redox status and past spatiotemporal isolation (due to the frozen state in the deeper samples).

### Sequence divergence from known functional genes

Our current understanding of functional gene diversity is largely limited by constraints on current primer coverage (Penton et al., 2013a). In most cases this artificially limits the retrieved diversity of the N-gene-harboring microbial community. As an example, until recently and due to the lack of coverage of typical *nosZ* primers, an important atypical *nosZ* clade that potentially mediates soil N_2_O sink capacity was left undiscovered (Sanford et al., 2012; Jones et al., 2013, 2014). Additionally, increased recovery of more diverse (or even non-target) sequences is due to deeper sequencing projects that reveal the rare, but targeted taxa and shotgun metagenomics that results in a smaller number, but potentially more diverse sets of target genes. This is supported in that, for all N functional genes except NirK in Alaska, OTUs with a relative abundance >1.0% had significantly higher percent identities (92.2 ± 0.5%) to the reference database (*t*-test, *p* = 0.01) than the more rare OTUs (88.8 ± 0.7%).

While other genes shared similar OTU identities to their respective databases in both AK and OK, NirK identities were markedly lower in the permafrost at an average 36.5% amino acid identity. Also, 26.2% of AK NirK sequences could not be matched to the reference database that contained only known NirK proteins. The high divergence of *nirK* compared explicitly to *nirS* was first shown using antibodies against *cd*_1_ *nirS* and Cu *nirK*. It was postulated that *nirK* originated earlier than *nirS*, with the resulting variation from a higher cumulative divergence (Coyne et al., 1989). The unmatched sequences are possibly due to (a) the presence of novel sequences not yet identified through metagenomic or targeted sequencing (b) reference database coverage limitation, or (c) mis-priming leading to the capture of non-nirK sequences. Mis-priming is unlikely as the permafrost samples were amplified in concert with the tallgrass prairie samples whose sequences averaged 81.7% identity to the reference database. To further investigate we BLAST'd the reference protein sequences against the NCBI reference databases. We found that, in some cases, sequences more closely matched putative environmental nirK sequences with as high as 79% identity (e.g., nirK sequences obtained from soils in Finland, Braker et al., 2012). When compared against an archaeal nirK variant (54d9) (Treusch et al., 2005), alignment showed a maximum 29% identity to our representative sequences. As with our sequences, the Treusch archaeal nirK variant also exhibited low identity (25–32%) to reference nirK sequences. Overall, this indicates the likely presence of a poorly-understood *nirK* harboring community in the permafrost samples, possibly from spatial and temporal isolation due to the frozen state maintained from the Pleistocene era.

Methodology is a central issue to many molecular microbial ecology studies as all current methods have their limitations. Primer based studies like this one have their advantages (sensitivity and a focus on functions of interest) but also disadvantages (primer bias and representative sampling, Zhou et al., 2011, 2013). In this study we employed widely used N-cycle primers, but with the expanding sequence database, it is apparent that primer coverage of diversity is limiting (Penton et al., 2013a). However, by keeping conditions constant, comparisons among treatments or conditions can be made reflecting information for the sampled group though it is not comprehensive for the guild. It is time though for the development of new, broader coverage primers for both sequencing and quantitation. Ideally, qPCR primer design should target amplification at the clade level to enable a higher resolution analysis of microbial functional groups. This would allow for response comparisons among specific clades, thus providing data regarding the sensitivity of sub-groups to experimental warming.

In conclusion, our study demonstrated that a decade of warming in a tallgrass prairie resulted in significant shifts in *nirK* and *nosZ* harboring bacterial community structures as well as significantly higher richness and evenness of the diazotrophic and *nirS* harboring communities. As might be expected the 1 year of permafrost soil warming and with colder temperatures limiting growth rates, there was not much shift in N-processing communities. The fact that the *nirK* harboring permafrost community was significantly impacted with an accompanying decrease in richness is interesting, and perhaps relates to the very novel sequences and by inference novel populations that may be adapted to lower temperature responses. It is also notable that across sites, the *nirK* harboring communities were the most impacted by warming. As these sites are part of long-term studies, it will be important to compare the types and rates of response after equal time periods, and if these can be related to process changes. As such, the AK information here provides a beginning reference for such a long-term comparison.

### Conflict of interest statement

The Guest Associate Editor Stuart Findlay declares that despite having hosted a Frontiers Research Topic with the Review Editor Jérôme Comte, the review process was handled objectively and no conflict of interest exists. The authors declare that the research was conducted in the absence of any commercial or financial relationships that could be construed as a potential conflict of interest.
