# Supplementary material for: Denitrifying and diazotrophic community responses to artificial warming in permafrost and tallgrass prairie soils
Source: Front Microbiol. 2015 Jul 21;6:746. doi: 10.3389/fmicb.2015.00746 (PMC4523034; doi:10.3389/fmicb.2015.00746)
Supplement: Supplementary file 1 [file DataSheet1.DOCX]

**Denitrifying and diazotrophic community responses to artificial warming in permafrost and tallgrass prairie soils**

C. Ryan Penton^1,2*^ , Derek St. Louis^1^, Amanda Pham^1^, James R. Cole^1^, Liyou Wu^3^, Yiqi Luo^4^, E.A.G. Schuur^4^, Jizhong Zhou^3,6,7^, James M. Tiedje^1^

^1^ Center for Microbial Ecology, Michigan State University, East Lansing, MI

^2^ College of Letters and Sciences, Arizona State University, Polytechnic Campus, Mesa, AZ

^3^ Institute for Environmental Genomics and Department of Microbiology and Plant Biology, University of Oklahoma, Norman, OK

^4^ Department of Microbiology and Plant Biology, University of Oklahoma, Norman, OK

^5^ Center for Ecosystem Science and Society, Department of Biological Sciences, Northern Arizona University, Flagstaff, AZ

^6^ State Key Joint Laboratory of Environment Simulation and Pollution Control, School of Environment, Tsinghua University, Beijing 100084, China

^7^ Earth Science Division, Lawrence Berkeley National Laboratory, Berkeley, CA, USA

***Correspondence:** Dr. C. Ryan Penton, Arizona State University Polytechnic Campus, College of Letters and Sciences, Mail Code 2780, Arizona State University, 6073 S Backus Mall, Mesa, AZ, 85212

crpenton@asu.edu.

**Supplementary Table 1**. Primers and PCR conditions for functional gene pyrosequencing.

| **Functional Group** | **Primers** | **Sequence (5'-3')** | **PCR conditions** | **Reference** |
| --- | --- | --- | --- | --- |
| *nirS* | cd3aF | GTS AAC GTS AAG GAR ACS GG | 94 °C -5 min, 30 x [94°C for 30 s, 51°C -1 min, 72°C -1 min], 72°C – 7 min | Michotey et al. 2000  Thröback et al., 2004 |
|  | R3cd | GAS TTC GGR TGS GTC TTG A |  |  |
| *nirK* | 517F | TTY GTS TAY CAC TGC GCV CC | 94 °C -5 min, 15 x [94°C for 45 s, 57°C^1^ -1 min, 72°C -1 min], 25 x [94°C for 45 s, 47°C -1 min, 72°C -1 min], 72°C – 7 min | Chen et al., 2010 |
|  | 1055R | GCY TCG ATC AGR TTR TGG TT |  |  |
| *nosZ* | nosZF | CGC TGT TCI TCG ACA GYC AG | 94 °C -3 min, 10 x [94°C for 45 s, 64°C -1 min, 72°C -1 min], 25 x [94°C for 45 s, 55°C -1 min, 72°C -1 min], 72°C – 7 min | Rich et al., 2003 |
|  | nosZR | ATG TGC AKI GCR TGG CAG AA |  |  |
| *nifH* | PolF | TGC GAY CCS AAR GCB GAC TC | 94 °C - 3 min, 30 x [94°C – 45 s, 62°C – 45 s, 72°C for 1 min], 72°C – 7 min | Poly et al., 2001  Wang et al., 2013 |
|  | PolR | ATS GCC ATC ATY TCR CCG GA |  |  |

**Supplementary Table 2**. Sequence processing results.

|  | **Quality Filtered** | **# Samples** | **% Pass Framebot** | **Reads/**  **Sample** | **Total Reads** | **# OTUs** | |
| --- | --- | --- | --- | --- | --- | --- | --- |
|  |  |  |  |  |  | **Fwd** | **Rev** |
| *nifH* | 144,385 | 23 | OK: 99.2%  AK: 99.7% | 3800 | 87,400 | 231 | |
| *nirS* | 272,345 | 22 | OK: 65.3%  AK: 58.6% | 1195^1^ | 52,580 | 601 | 644 |
| *nirK* | 315,006 | 25 | OK: 95.6%  AK: 94.9% | 1600^1^ | 80,000 | 828 | 862 |
| *nosz* | 212,931 | 27 | OK: 75.8%  AK: 70.5% | 1053^1^ | 56,862 | 456 | 688 |

^1^ The number of reads/sample were parsed equally into forward and reverse reads due to the requirement for bi-directional sequencing before analyses.

**Supplementary Table 3**. Averages of Alaskan soil bulk density (BD), percent nitrogen (%N) and percent carbon (%C) with standard deviations between the organic and mineral layers. Superscript letters indicate ANOVA grouping with Tukey’s correction.

|  | Organic | | Mineral | |
| --- | --- | --- | --- | --- |
|  | Control | Warming | Control | Warming |
| BD | 0.35 ± 0.26 ^A^ | 0.17 ± 0.14 ^A^ | 0.91 ± 0.41 ^B^ | 0.62 ± 0.26 ^B^ |
| % N | 1.28 ± 0.41 ^A^ | 1.33 ± 0.34 ^A^ | 0.32 ± 0.21 ^B^ | 0.44 ± 0.36 ^B^ |
| % C | 34.89 ± 8.59 ^A^ | 38.33 ± 8.29 ^A^ | 9.04 ± 5.89 ^B^ | 10.4 ± 7.88 ^B^ |

**References**

Chen Z., Luo X., Hu, R., Wu, M., Wu, J., Wei, W. (2010) Impacts of long-term fertilization on the composition of denitrifier communities based on nitrite reductase analyses in a paddy soil. *Microb. Ecol.* 60, 850-861.

Michotey, V., Mejean, V., Bonin, P. (2000) Comparison of Methods for Quantification of Cytochrome cd1-Denitrifying Bacteria in Environmental Marine Samples. *Appl. Environ. Microbiol.* 66, 1564-1571.

Poly, F., Monrozier, L. J., Bally, R. (2001a). Improvement in the RFLP procedure for studying the diversity of nifH genes in communities of nitrogen fixers in soil. *Res. Microbiol.* 152, 95-103.

Rich, J. J., Heichen, R. S., Bottomley, P. J., Cromack, K., Myrold, D. D. (2003) Community composition and functioning of denitrifying bacteria from adjacent meadow and forest soils. *Appl. Environ. Microbiol.* 69, 5974–5982.

Thröback, I. N., Enwall, K., Jarvis, A., Hallin, S. (2004) Reassessing PCR primers targeting nirS, nirK and nosZ genes for community surveys of denitrifying bacteria with DGGE. *FEMS Microbiol. Ecol.* 49, 401-417.

Wang, Q., Quensen, J. F., Fish, J. A., Lee, T. K., Sun, Y., Tiedje, J. M., Cole, J. R. (2013) Ecological patterns of *nifH* genes in four terrestrial climatic zones explored with targeted metagenomics using FrameBot, a new informatics tool. *mBIO* 4(5):e00592-13.
