# Supplementary figures and images for: Denitrifying and diazotrophic community responses to artificial warming in permafrost and tallgrass prairie soils
Source: Front Microbiol. 2015 Jul 21;6:746. doi: 10.3389/fmicb.2015.00746 (PMC4523034; doi:10.3389/fmicb.2015.00746)

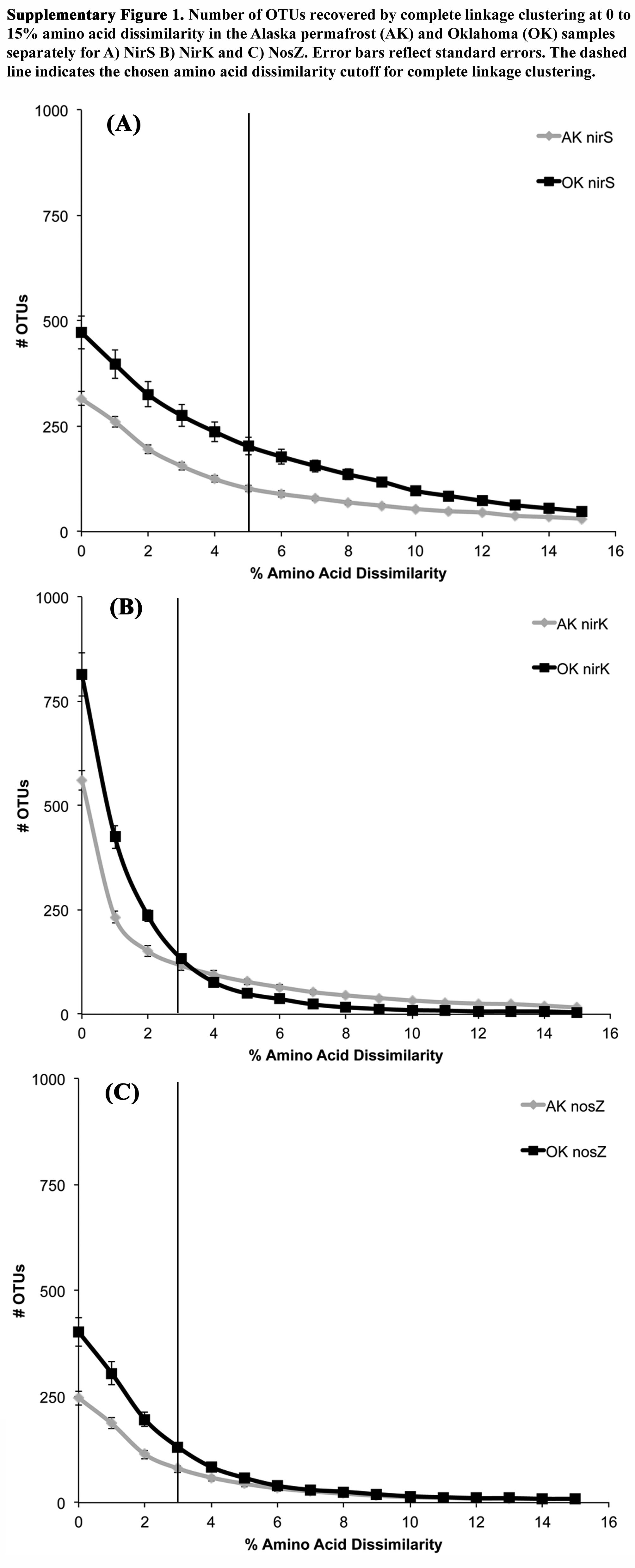

Supplement: Supplementary file 2 [file Image1.TIF]

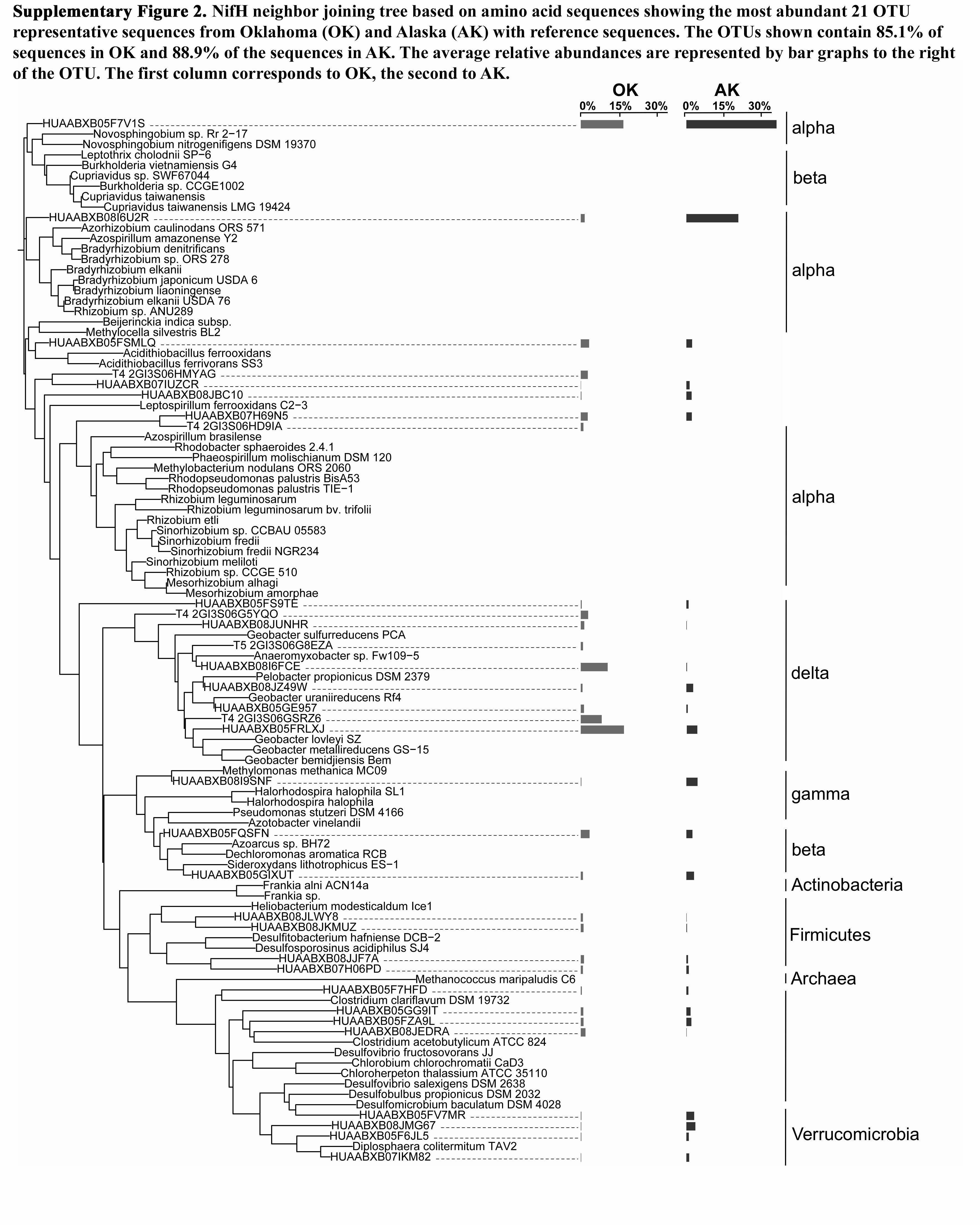

Supplement: Supplementary file 3 [file Image2.TIF]

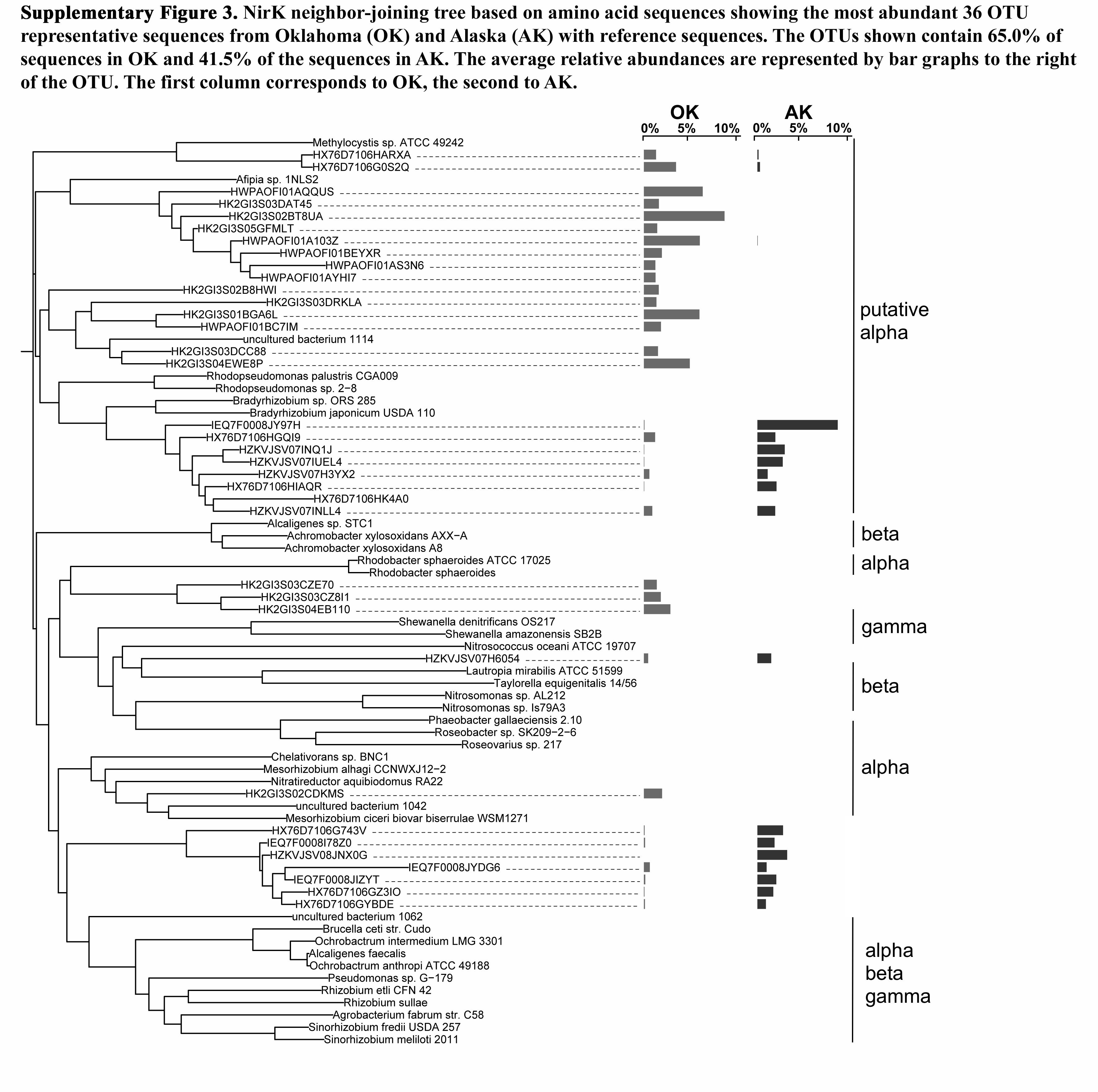

Supplement: Supplementary file 4 [file Image3.TIF]

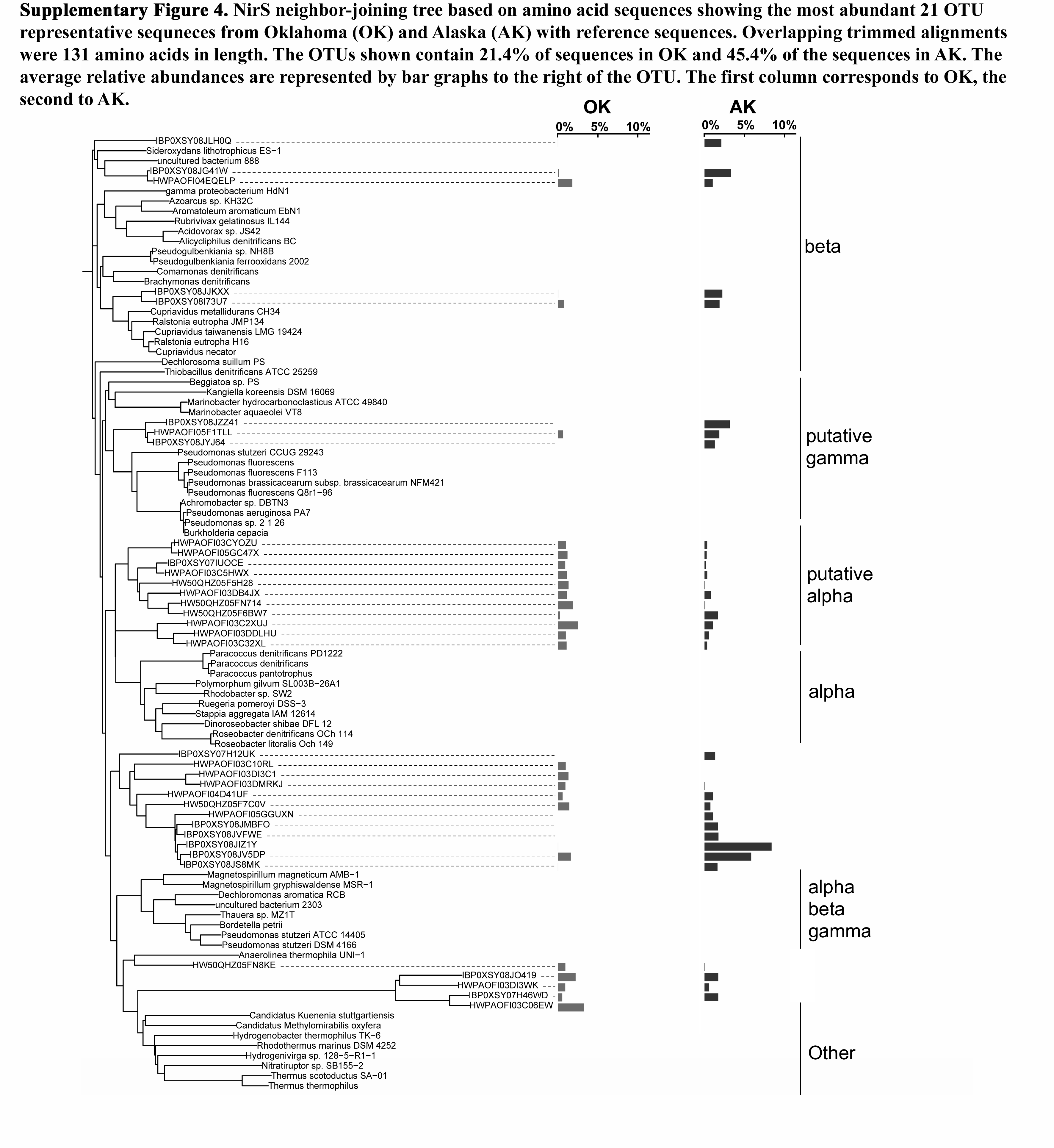

Supplement: Supplementary file 5 [file Image4.TIF]

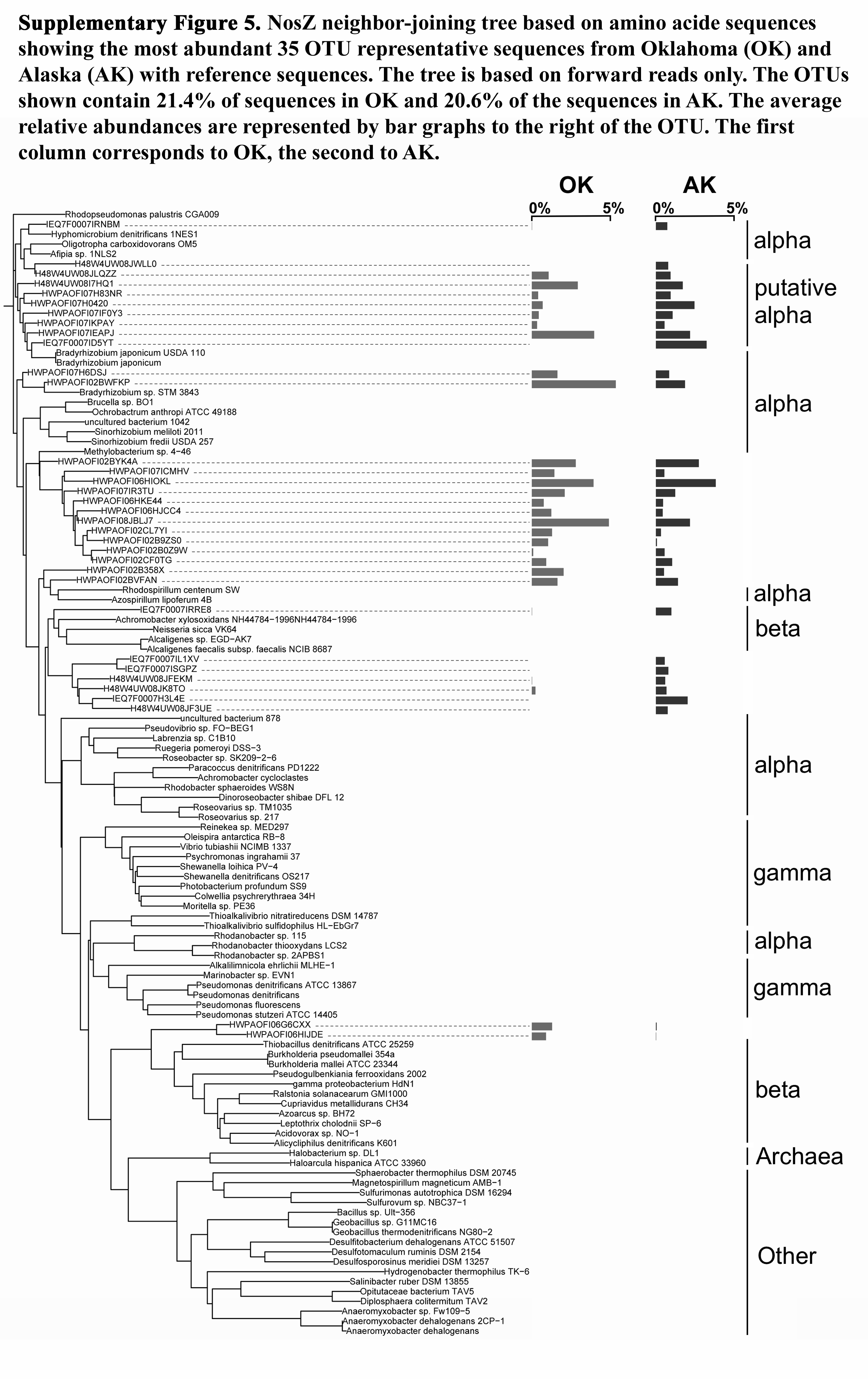

Supplement: Supplementary file 6 [file Image5.TIF]
